# Genome-wide analysis of the glutathione S-transferase family in *Vaccinium corymbosum* and functional characterization of *VcGSTU53* in response to abiotic stress

**DOI:** 10.3389/fpls.2026.1761092

**Published:** 2026-03-12

**Authors:** Youwen Tian, Bowei Wang, Jinjie Xing, Jingyu Wu, Limei Che, Qiyao Zhang, Yadong Li, Li Chen, Haiyue Sun

**Affiliations:** 1 College of Horticulture, Jilin Agricultural University, Changchun, Jilin, China; 2 Shijiazhuang Institute of Pomology, Hebei Academy of Agriculture and Forestry Sciences, Shijiazhuang, Hebei, China; 3 Jilin Provincial Agro-Tech Extension Center, Changchun, Jilin, China

**Keywords:** *Vaccinium corymbosum*, genome-wide, glutathione S-transferases (GSTs), abiotic stress, aluminum stress

## Abstract

**Introduction:**

As a high-value small fruit crop worldwide, blueberry is prized for its health-promoting properties. Glutathione S-transferases (GSTs) are a large and complex family of enzymes that play vital roles in flavonoid metabolism, plant growth and development, and responses to abiotic stress. However, relatively little is known about the GST gene family in blueberry.

**Methods:**

In this study, genome-wide identification and bioinformatics analysis of the blueberry GST gene family were conducted. The candidate gene *VcGSTU53* was further analyzed for its response to abiotic stress using transcriptome profiling, subcellular localization, and heterologous expression assays.

**Results:**

A total of 190 *VcGST* genes were identified and classified into 14 subclasses: tau (U, 75), phi (F, 28), EF1G (17), lambda (L, 10), MAPEG (10), zeta (Z, 8), GHR (8), Metaxin (M, 7), GST2N (6), DHAR (5), hemerythrin (H, 4), mPGES2 (4), TCHQD (4) and theta (T, 4); however, no iota (I) class GST was found. The lengths of the *VcGST* genes ranged from 366 bp to 4386 bp, encoding polypeptides of 121 to 1461 amino acids, with predicted molecular weights ranging from 13.86 kDa to 163.02 kDa, and theoretical isoelectric points ranging from 4.77 to 9.56. Approximately 46.32% of VcGST proteins were predicted to localize in the cytoplasm, with the remainder predicted to localize in the endomembrane system, chloroplasts, nucleus, mitochondria, extracellular space, and plasma membrane. Based on a comparative transcriptome profiling of 30 AlCl_3_-treated root samples from two cultivars, *VcGSTU53* was screened as a key aluminum-stress-responsive gene. VcGSTU53 encodes a protein of 230 amino acids, is localized in the cytoplasm, and exhibits transferase activity. Heterologous expression of *VcGSTU53* in *Escherichia coli* did not inhibit bacterial growth, but enhanced tolerance to aluminum stress. Notably, it also increased bacterial resistance to drought and cadmium stresses.

**Conclusion:**

These findings contribute to a deeper understanding of the functions of the GST family in plants under abiotic stress, which is of great significance for the development of new stress-resistant blueberry varieties.

## 1 Introduction

Glutathione S-transferases (GSTs; EC 2.5.1.18) constitute a family of multifunctional enzymes encoded by a large supergene family widely expressed in various organisms, and carry out a variety of physiological functions (Sheehan et al., 2001; Ashrafian and Bogle, 2004; Dixon et al., 2010). According to the gene structure, protein sequence similarity, gene function, and immunological characteristics within the family, Lallement et al. classified plant GSTs into 14 types, namely phi (F), tau (U), theta (T), zeta (Z), lambda (L), metaxin (M), hemerythrin (H), iota (I), dehydroascorbate reductase (DHAR), tetrachloro-hydroquinone dehalogenase (TCHQD), elongation factor 1Bγ (EF1Bγ), glutathionyl hydroquinone reductase (GHR), GSTs with two thioredoxins (GST2N), and microsomal prostaglandin E synthase type 2 (mPGES2) (Lallement et al., 2014; Sylvestre-Gonon et al., 2019; Cao et al., 2022). There are great sequence differences between different subfamilies of the GST supergene family, but the overall structure of the GST protein is highly conserved. GSTs contain an N–terminal domain and a C–terminal domain. The N–terminal domain includes a catalytic residue for glutathione (GSH) binding and catalysis, whereas the less conserved C–terminal domain binds hydrophobic substrates and determines GST specificity and activity (Wei et al., 2019).

GSTs were first discovered in animals as a result of their importance in the metabolism and detoxification of drugs (Wilce and Parker, 1994). Plant GSTs were identified in *Zea mays* (maize) for the first time during studies on herbicide detoxification and have since been widely investigated (Shimabukuro et al., 1970). In plants, GST proteins are essential for normal development and physiological functions. Studies have shown that GST proteins reduce toxic damage to plants through conjugation, binding and compartmentalization stages (Edwards and Dixon, 2005; Vaish et al., 2020). GST proteins can enhance the ability of plants to adapt to stress and are involved in the degradation of pesticides and herbicides (Hayes et al., 2005; Csiszár et al., 2014). In addition, plant GSTs play important roles responses to biotic and abiotic stresses, such as heavy metals (Gunning et al., 2014; Gao et al., 2020; Jing et al., 2021), cold (Kayum et al., 2018; Song et al., 2021), salinity (Sharma et al., 2014; Xu et al., 2016), pathogen attack (Sappl et al., 2009; Shen et al., 2015), herbicide detoxification (Chronopoulou et al., 2012), and in responses to high temperature (Islam et al., 2019), light (Jiang et al., 2010), and hormonal signals (Yamaguchi-Shinozaki and Shinozaki, 2005). However, the detailed mechanisms are currently unclear. With advances in genome sequencing and genome-wide analyses, GST gene families have been widely identified in numerous plant species, including *Oryza sativa* (79), *Arabidopsis thaliana* (55), *Malus domestica* (38), and *Solanum lycopersicum* (90), among others (Zhao et al., 2021; Jain et al., 2010; Xu et al., 2017; Han et al., 2018; Wang et al., 2018; Wang et al., 2020; Zhuge et al., 2020; Lai et al., 2021). Although the GST gene family has been extensively characterized in various plant species, its exploration has been limited in *Vaccinium* spp. (blueberry).

Blueberry is a shrub plant belonging to the Ericaceae family (Becker et al., 2024). In recent years, blueberry cultivation is almost ubiquitous worldwide due to the rising demand (Lachowicz-Wiśniewska et al., 2024). Blueberries are popular with consumers around the world because of their high nutritional value. The fruit has numerous beneficial effects for humans (Stull et al., 2024). Its active constituents can promote retinoid re-synthesis and improve immunity, as well as having anti-inflammatory, anti-cardiovascular disease, antiaging, and anticancer effects, which greatly promote human health (Silva et al., 2020). Consumers consider blueberries “superfoods” because they contain high concentrations of antioxidants (Skrovankova et al., 2015). In this study, the public reference genome sequence (Colle et al., 2019) of blueberry were used to identify a total of 190 GST members. The classfication, chromosomal locations, structural features, and evolutionary divergence of the VcGST gene family were characterized. Subsequently, the expression profiles of *VcGST* genes induced by aluminum stress were analyzed, and *VcGSTU53* was further studied as a candidate gene for aluminum response. Subcellular localization and *in vitro* enzyme activity assays confirmed that VcGSTU53 is localized in the cytoplasm and exhibits transferase activity with glutathione (GSH) and 1-chloro-2, 4-dinitrobenzene (CNDB) as substrates. Heterologous expression of *VcGSTU53* in *Escherichia coli* enhances its tolerance to abiotic stresses such as aluminum, cadmium and drought. These insights will advance our understanding of GST-mediated aluminum tolerance mechanisms and provide a valuable reference for molecular breeding strategies aimed at improving horticultural species resilience to abiotic stresses.

## 2 Materials and methods

### 2.1 Identification and characterization of glutathione S-transferases in blueberry

Genome sequences and annotation information of *Vaccinium corymbosum* (highbush blueberry) cultivar ‘Draper’ were obtained from the GDV database (https://www.vaccinium.org/analysis/49), based on genome assembly v1.0 (Colle et al., 2019). A total of 3, 884 glutathione S-transferases (GST) protein sequences from more than 50 species, including *Arabidopsis thaliana*, *Vitis vinifera*, *Malus domestica*, and *Brassica oleracea*, were used as query sequences (**Supplementary Table 1**) to identify VcGST proteins using the BLASTP algorithm with an E-value cutoff of ≤ 1e -5. Hidden Markov model (HMM) profiles for the GST_N domain (Pfam IDs: PF02798, PF13409, PF13417, PF17172, and PF18485) and GST_C domain (Pfam IDs: PF00043, PF13410, PF14497, PF14834, PF16865, PF17171, PF22041, and PF22119) from the Pfam database (https://www.ebi.ac.uk/interpro/entry/pfam) were obtained (Paysan-Lafosse et al., 2025). These profiles were used to identify VcGST protein candidates using HMMER3.0 with default settings (Yang et al., 2024). Then, the Conserved Domain Database (CDD) of NCBI (https://www.ncbi.nlm.nih.gov/Structure/cdd/wrpsb.cgi) was used to validate the conserved domains of these candidate sequences (Marchler-Bauer et al., 2015).

The ProtParam tool on the ExPASy server (https://web.expasy.org/protparam) was used to analyze the physicochemical properties of the VcGST proteins (Wilkins et al., 1999), including sequence length, molecular weight (MW), theoretical isoelectric point (pI), instability index (II), aliphatic index (AI), and grand average of hydropathicity (GRAVY). The Cell-PLoc 2.0 (http://www.csbio.sjtu.edu.cn/bioinf/Cell-PLoc-2) was used to predict subcellular localizations of VcGSTs (Chou and Shen, 2008).

### 2.2 Phylogenetic tree construction and nomenclature of VcGSTs

To determine the evolutionary relationships of GST proteins, multiple sequence alignment was performed using MEGA 11 with GST protein sequences from *Vaccinium corymbosum*, *Arabidopsis thaliana*, *Vitis vinifera*, and *Brassica oleracea* (Tamura et al., 2021). A phylogenetic tree was constructed using the maximum likelihood (ML) method with 1, 000 bootstrap replicates, and the Jones-Taylor-Thornton (JTT) model was applied (Jones et al., 1992). All other parameters were set to default values.

The nomenclature of blueberry GSTs followed the method previously proposed by Dixon et al. (Dixon et al., 2002). For example, VcGSTU, VcGSTF, VcEF1G, VcGSTL, VcMAPEG, VcGHR, VcGSTZ, VcGSTM, VcGST2N, VcDHAR, VcGSTH, VcGSTT, VcmPGES2, VcTCHQD correspond to tau, phi, EF1G, lambda, MAPEG, GHR, zeta, metaxin, GST2N, DHAR, hemerythrin, theta, mPGES2, and TCHQD classes, respectively. The numbers signify the different members of the subfamily.

### 2.3 Chromosomal location and collinearity analysis of VcGSTs

Highbush blueberry cultivar ‘Draper’ is a tetraploid species with a total of 48 chromosomes. In the genome sequence, the 48 longest scaffolds were assigned to chromosomes 1 to 48. Gene location and density information were obtained using GFF file of blueberry, and the chromosomal location of *VcGSTs* was visualized utilizing TBtools-II software “Gene Location Visualize (Advanced)” function (Chen et al., 2023).

Syntenic relationships among *Vaccinium corymbosum*, *Arabidopsis thaliana*, and *Vitis vinifera* were analyzed and visualized using the “One Step MCScanX” and “Multiple Synteny Plot” functions in TBtools-II with default settings. Additionally, the synteny within the blueberry genome was assessed using the “Dual Synteny Plot” function in TBtools-II (Wang et al., 2012; Chen et al., 2023; Wang et al., 2024). Additionally, the ratio of nonsynonymous substitution rate (Ka) to synonymous substitution rate (Ks) for syntenic gene pairs (Ka/Ks) was calculated using the “Simple Ka/Ks Calculator” function in TBtools. This ratio can be used to predict whether the genes are under selective pressure (Zhang, 2022).

### 2.4 Analysis of the conserved motif, gene structure, and cis-acting elements

A phylogenetic tree of the GST family in blueberry was constructed using maximum likelihood (ML) with a bootstrap value of 1000. The MEME online program (v5.5.1, https://meme-suite.org/meme) was used to analyze the 20 conserved motifs of VcGST proteins (Bailey et al., 2015). The NCBI-CDD was used to analyze conserved domains. Exon-intron organization data were obtained from the GFF file. A comprehensive figure, including the phylogenetic tree, motif composition, conserved domains, and gene structure, was generated using the “Advanced Circos” function in TBtools (Chen et al., 2023).

The 2000 bp upstream sequences of each gene coding sequence (CDS) were extracted as the promoter regions. The PlantCARE database (http://bioinformatics.psb.ugent.be/webtools/plantcare/html) was used to scan and predict cis-acting elements within these promoter sequences (Lescot et al., 2002). The distribution of the predicted cis-acting elements on the promoters was visualized using the “Simple BioSequence Viewer” function in TBTools (Chen et al., 2023).

### 2.5 Comparative transcriptomic profiling of VcGSTs under aluminum stress

Aluminum stress treatment was performed using a hydroponic system in an artificial climate chamber. The experimental materials were six-month-old softwood cuttings of two blueberry cultivars, ‘Duke’ and ‘Draper’, sourced from the Small Berry Genetic Breeding Center of Jilin Agricultural University in Changchun, China (43°47′N, 125°24′E). The plants were exposed to a gradient of Al^3+^ concentrations (0, 200, 400, 800, and 1600 μM, supplied as AlCl_3_·6H_2_O) for 80 hours. Each treatment included three biological replicates, with eight plants per replicate. Thirty root samples were collected, immediately frozen in liquid nitrogen, and stored at -80°C. Total RNA was extracted, and transcriptome sequencing was performed using the BGI-T7 platform (unpublished data). Differential expression analysis of *VcGST* genes was conducted based on fragments per kilobase of transcript per million mapped reads (FPKM) values, with the thresholds of |log_2_(fold change)| ≥ 1 and an adjusted P-value (FDR) < 0.05. A heatmap was generated to visualize gene expression patterns using the OmicStudio tools (https://www.omicstudio.cn).

### 2.6 Cloning of VcGSTU53 and subcellular localization

Total RNA extracted from blueberry roots was reverse-transcribed into first-strand cDNA. The complete coding sequence of the *VcGSTU53* gene (*VaccDscaff35-augustus-gene-220.36*) was cloned using specific primers (**Supplementary Table 2**). For subcellular localization analysis of VcGSTU53, the plasmids of pGDG-VcGSTU53-mGFP and the empty vector (pGD-mGFP) were introduced into *Agrobacterium tumefaciens* EHA105 by the liquid nitrogen quick-freezing method and then transfected into tobacco leaves (Goodin et al., 2002; Sparkes et al., 2006). The injected tobacco plants were cultured under dark conditions for 48 hours, and fluorescence signals were detected using a Leica SP8 laser confocal microscope (Leica, Wetzlar, Germany). The pGD-mGFP vector was provided by Professor Wenxian Sun from the College of Plant Protection, Jilin Agricultural University.

### 2.7 Prokaryotic expression, purification and enzyme activity of VcGSTU53

The coding sequence of *VcGSTU53* gene was cloned into the pET28a(+) vector to construct the fusion expression vector pET28a-*VcGSTU53* with an N-terminal His-tag. The recombinant plasmid harboring the target gene was transformed into *Escherichia coli* BL21(DE3) competent cells. Positive clones were selected and inoculated into Luria-Bertani (LB) liquid medium containing 30 µg/mL kanamycin, and cultured at 37°C until the OD_600_ reached approximately 0.6. Protein expression was induced by adding 0.5 mM isopropyl β-D-1-thiogalactoside (IPTG) and incubated at 20°C overnight or at 37°C for 6 hours to optimize expression conditions. An uninduced culture was used as a negative control (Rosano and Ceccarelli, 2014). Cells were harvested by centrifugation, lysed by sonication, and the supernatant was collected as crude protein. The crude protein was purified using nickel-nitrilotriacetic acid (Ni-NTA) affinity chromatography. The column was equilibrated, followed by sample loading, incubation, washing, and elution steps. The purified protein was dialyzed against 50 mM Tris, 300 mM NaCl, pH 8.0, concentrated using PEG20000, filtered through a 0.45 µm membrane, aliquoted into 1 mL tubes, and stored at –80°C. Protein purity and molecular weight were assessed by sodium dodecyl sulfate-polyacrylamide gel electrophoresis (SDS-PAGE) on a 12% gel. The recombinant protein was confirmed by western blot analysis using mouse anti-His tag primary antibody and goat anti-mouse secondary antibody.

The protein content was detected using a BCA Protein Concentration Assay Kit (PC0020, Solarbio, Beijing, China). The 250 μL assay mixture consisted of 200 μM protein, 1×PBS buffer (pH 6.8), 1 mM glutathione (GSH), 1 mM dehydroascorbic acid (DHA), and 1-chloro-2, 4-dinitrobenzene (CDNB). The same mixture containing the target protein inactivated by boiling served as the control (Habig et al., 1974). These analyses were conducted in triplicate. After 30 min reaction time, the absorbance of the mixture at 340 nm was determined with a UV spectrophotometer. The Michaelis constant (K_m_) and maximum velocity (V_max_) were determined by fitting the initial velocity data to the Michaelis–Menten equation using nonlinear regression analysis in Origin software (OriginLab Corporation, Northampton, MA, USA).

### 2.8 Growth kinetics of E. coli expressing VcGSTU53 in medium under various stress conditions

Growth kinetics were analyzed under various stress conditions. After induction with 0.5 mM IPTG at 20°C until OD600 reached 0.6, cultures of the recombinant strain BL21/pET28a-VcGSTU53 and the empty vector control strain BL21/pET28a were diluted 100 fold into fresh medium. The diluted cultures were incubated at 37°C with shaking (200 rpm) for 24 hours. The OD_600_ was measured at 2 hours intervals to generate growth curves. The following stress conditions were applied simultaneously with dilution: aluminum stress (0.25, 0.5, 1, 2, and 4 mM AlCl_3_·6H_2_O), salt stress (500 mM NaCl), drought stress (1.2 M PEG8000), and cadmium stress (1 mM CdCl_2_).

## 3 Results

### 3.1 Identification and physicochemical characterization of VcGSTs family in blueberry

A total of 190 GST proteins containing GST_C or GST_N domains in the *Vaccinium* ‘Draper’ genome database; but some genes contained only one conserved domain because of the unique structure of their subfamily. According to genome annotation data, the lengths of the *VcGST* genes ranged from 366 bp to 4386 bp, encoding polypeptides with lengths of 121 to 1461 amino acids. The predicted molecular weight (MW) of the putative proteins ranged from 13.86 KDa to 163.02 KDa, and the theoretical isoelectric point ranged from 4.77 to 9.56. It was predicted that 46.32% of VcGST proteins localize in the cytoplasm, with the remaining predicted to localize in the endomembrane system, chloroplasts, nucleus, mitochondria, extracellular space, and plasma membrane (**Supplementary Table 3**).

### 3.2 Classification and phylogenetic relationships of GST proteins

A total of 190 VcGST proteins were identified and classified into 14 distinct subfamilies. The tau (U, 75 members; VcGSTU1–75) and phi (F, 28 members; VcGSTF1–28) subfamilies were the most abundant, together constituting over half of all VcGSTs, with the tau subfamily being the largest as commonly observed in higher plants. The remaining 12 subfamilies included EF1G (17 members), lambda (L, 10), MAPEG (10), zeta (Z, 8), GHR (8), Metaxin (M, 7), GST2N (6), DHAR (5), TCHQD (4), theta (T, 4), mPGES2 (4), and hemerythrin (H, 4). Notably, no members belonging to the iota (I) subfamily were identified in the blueberry genome.

**Figure 1:**
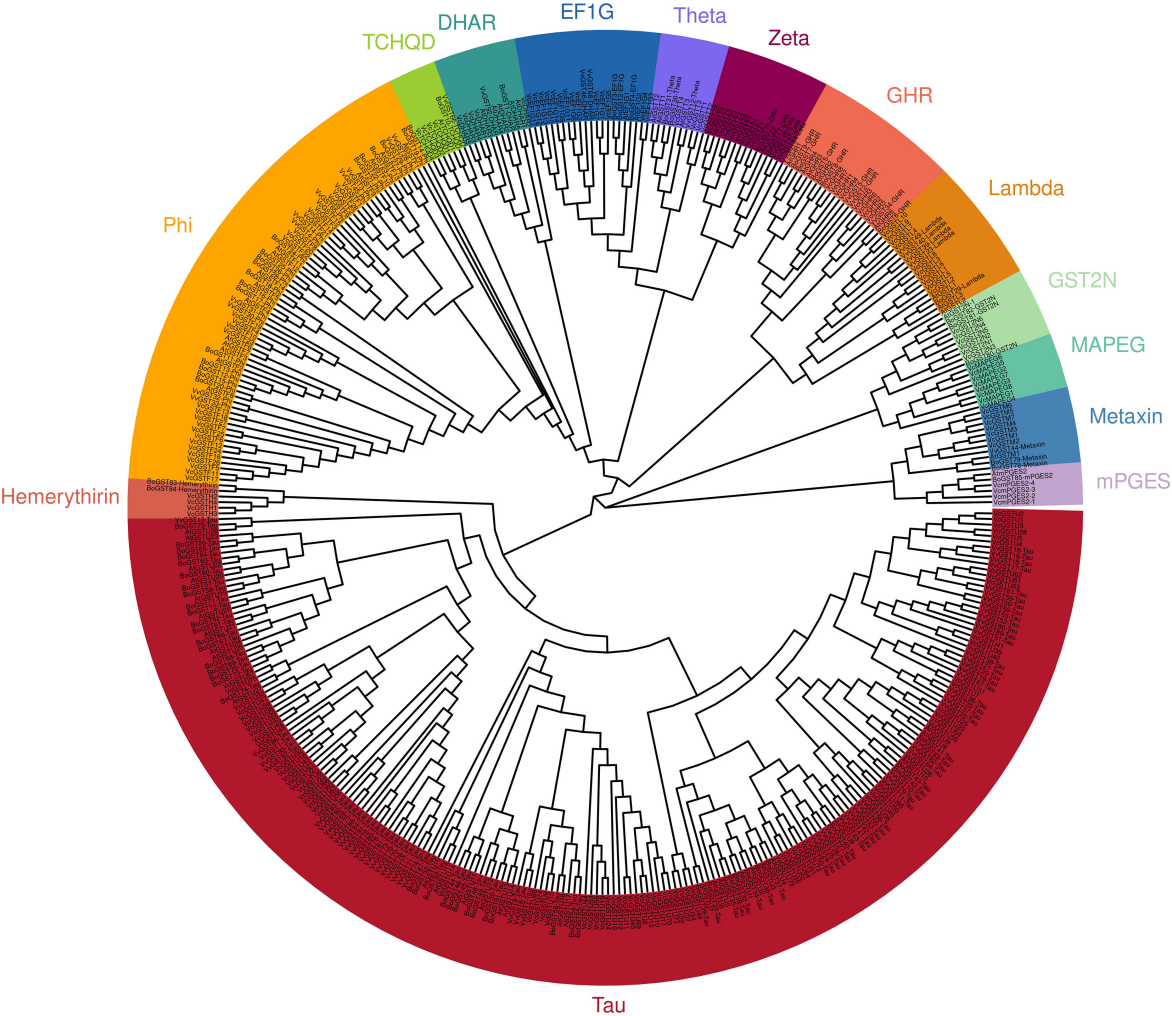
The phylogenetic tree of GST proteins from *Vaccinium corymbosum* (Vc), *Arabidopsis thaliana* (At), *Vitis vinifera* (Vv), and *Brassica oleracea* (Bo) was conducted using the maximum likelihood method (bootstrap = 1, 000).

To determine the evolutionary relationships of GSTs among *Vaccinium*, *Arabidopsis thaliana*, *Vitis vinifera*, and *Brassica oleracea*, a phylogenetic tree was constructed using their GST protein sequences. As shown in **Figure 1**, there was a high degree of conservation among GST protein subfamilies. All proteins in the same GST subfamily were clustered together and showed high homology. Based on branch length, the *Vaccinium* GSTs were most closely related to those of *Vitis vinifera*. The different species had different numbers of GSTs in the various subfamilies. The high homology of GSTs between blueberry and grape indicated a close genetic relationship.

### 3.3 Chromosomal location and collinearity analysis of VcGST genes

**Figure 2:**
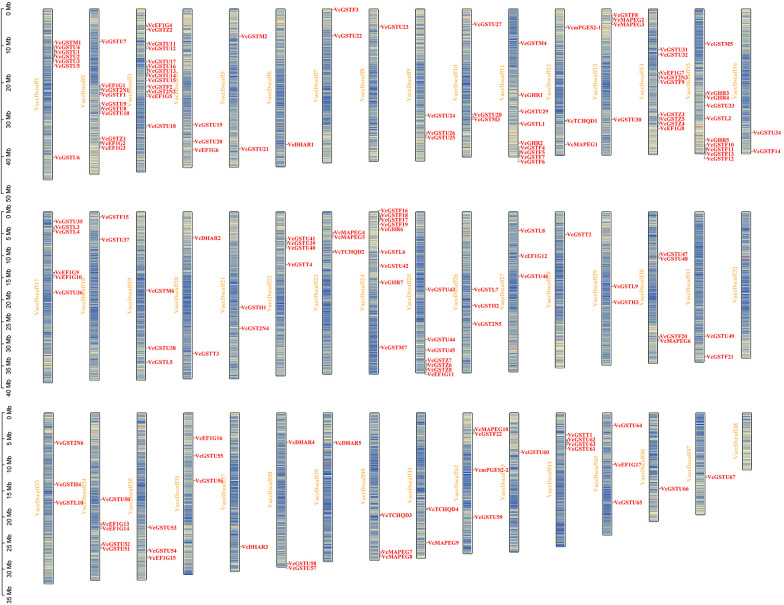
The chromosomal distribution of 190 *VcGST* genes in *Vaccinium corymbosum ‘Draper’* (2n = 4x = 48). Genes located on other scaffolds are not shown.

*Vaccinium corymbosum* ‘Draper’ is a tetraploid species with 48 chromosomes (2n = 4x = 48). A total of 190 *VcGST* genes were identified and unevenly distributed across the 48 chromosomes (**Figure 2**). Chromosome 3 contained the highest number of *VcGST* genes (13), followed by chromosome 2 (10), whereas no GST genes were detected on chromosomes 32 and 48. Additionally, 17 *VcGST* genes (*VcGHR8*, *VcGSTF23*, *VcGSTF24*, *VcGSTF25*, *VcGSTF26*, *VcGSTF27*, *VcGSTF28*, *VcGSTU68*, *VcGSTU69*, *VcGSTU70*, *VcGSTU71*, *VcGSTU72*, *VcGSTU73*, *VcGSTU74*, *VcGSTU75*, *VcmPGES2–3*, and *VcmPGES2–4*) were located on unanchored scaffolds and could not be assigned to specific chromosomes, which may be attributed to incomplete genome assembly or current limitations in sequencing and assembly strategies.

Segmental and tandem duplications play important roles in the expansion of plant gene families. Among the 190 identified VcGST genes, 150 were derived from whole-genome duplication (WGD) or segmental duplication events and were distributed across 46 chromosomes, including 41 members of the tau class and 14 members of the phi class. In addition, 27 VcGST genes were identified as tandem duplicates, 12 as proximal duplicates, and one as a dispersed duplicate (**Supplementary Table 4**). These results indicate that WGD or segmental duplication represents the primary driving force underlying the expansion of the VcGST gene family. The expansion of the *VcGST* gene family in blueberry may be attributed to its allopolyploid genome.

**Figure 3:**
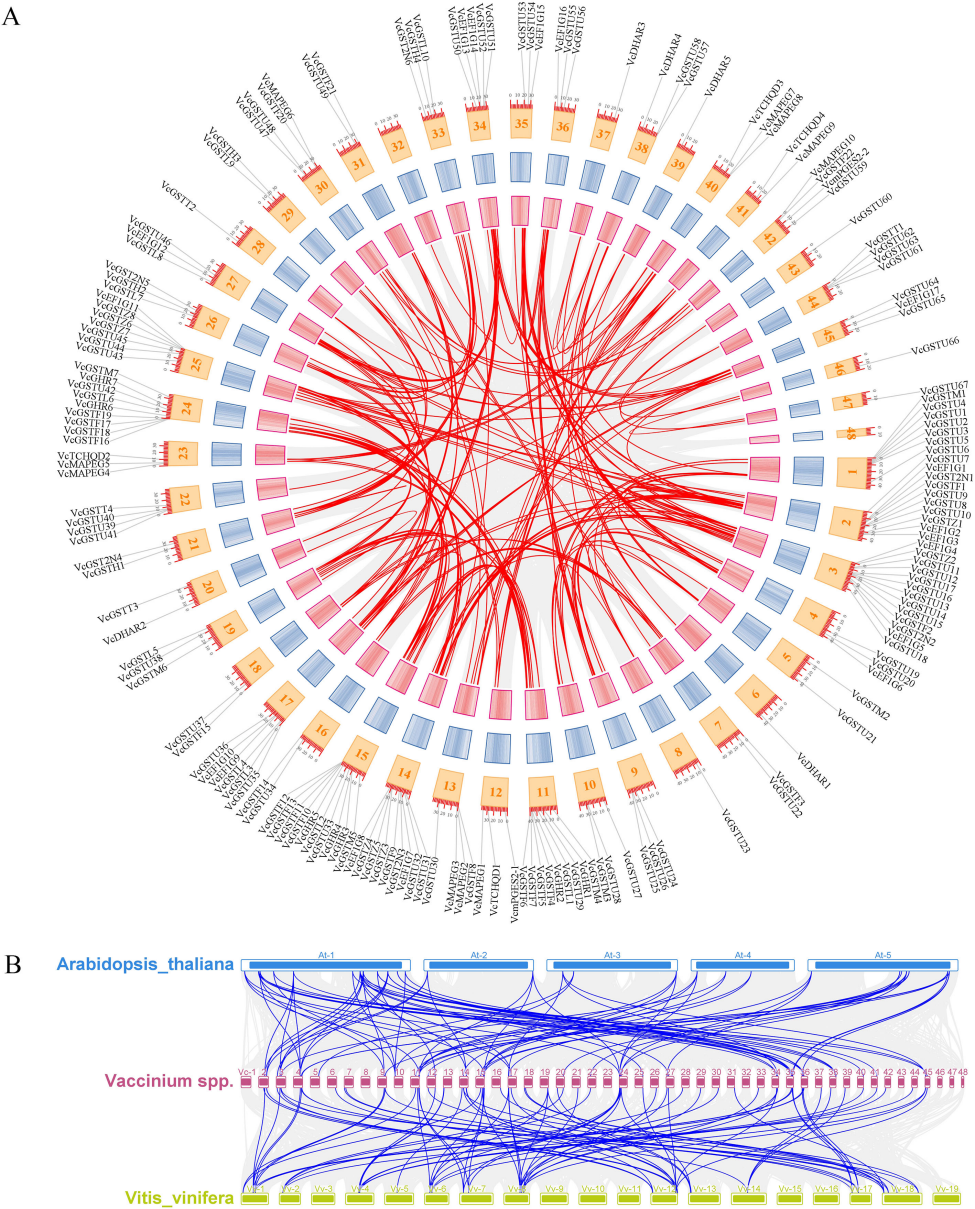
Synteny analysis of *VcGST* genes. **(A)** Intraspecies collinearity of *VcGST* genes within *Vaccinium corymbosum*. **(B)** Interspecies synteny of *VcGST* genes among *Vaccinium corymbosum*, *Arabidopsis thaliana*, and *Vitis vinifera*.

To further validate the contribution of WGD or segmental duplication to VcGST family expansion, collinearity analysis within the blueberry genome revealed that 137 VcGST genes were involved in 189 collinear gene pairs (**Figure 3A**). The ratio of the non-synonymous substitution rate (Ka) to the synonymous substitution rate (Ks) provides information about the type of selection pressure affecting gene pairs. The Ka/Ks ratio of collinear gene pairs ranged from 0 to 2.52. Only five GST collinear gene pairs (*VcGSTL7/VcGSTL10*, *VcGSTL7/VcGSTL9*, *VcGSTU20/VcGSTU26*, *VcEF1G15/VcEF1G16*, and *VcGSTL1/VcGSTL6*) had Ka/Ks ratios greater than 1, indicating that they have been under positive selection (**Supplementary Table 5**). In contrast, most duplicated *VcGST* gene pairs exhibited Ka/Ks ratios below 1, suggesting that they have been under purifying selection.

To further investigate the evolutionary mechanisms of the VcGST family, comparative synteny analyses were conducted between *Vaccinium corymbosum*. and two representative dicot species, *Arabidopsis thaliana* and *Vitis vinifera*. A total of 88 syntenic gene pairs were identified between blueberry and *A*. *thaliana*, involving 55 blueberry GST genes and 33 Arabidopsis GST genes. Similarly, 94 syntenic pairs were detected between blueberry and grapevine, comprising 80 blueberry and 30 grape GST genes (**Figure 3B**; **Supplementary Table 6**). These collinear relationships were distributed across 11 GST subfamilies, with GSTU, GSTF, and EF1G accounting for the largest numbers of members. The prevalent “many-to-one” synteny pattern suggests that segmental and tandem duplications contributed to the expansion of the blueberry GST gene family. Notably, 42 blueberry GST genes exhibited conserved collinearity in both Arabidopsis and grape, and the majority belonged to the tau, EF1G, and GHR subfamilies. This result indicates that these GST genes have retained stable genomic positions across divergent dicot lineages and represent evolutionarily conserved components of the blueberry GST gene family.

### 3.4 Conserved motifs, domains, and gene structures of VcGST genes

**Figure 4:**
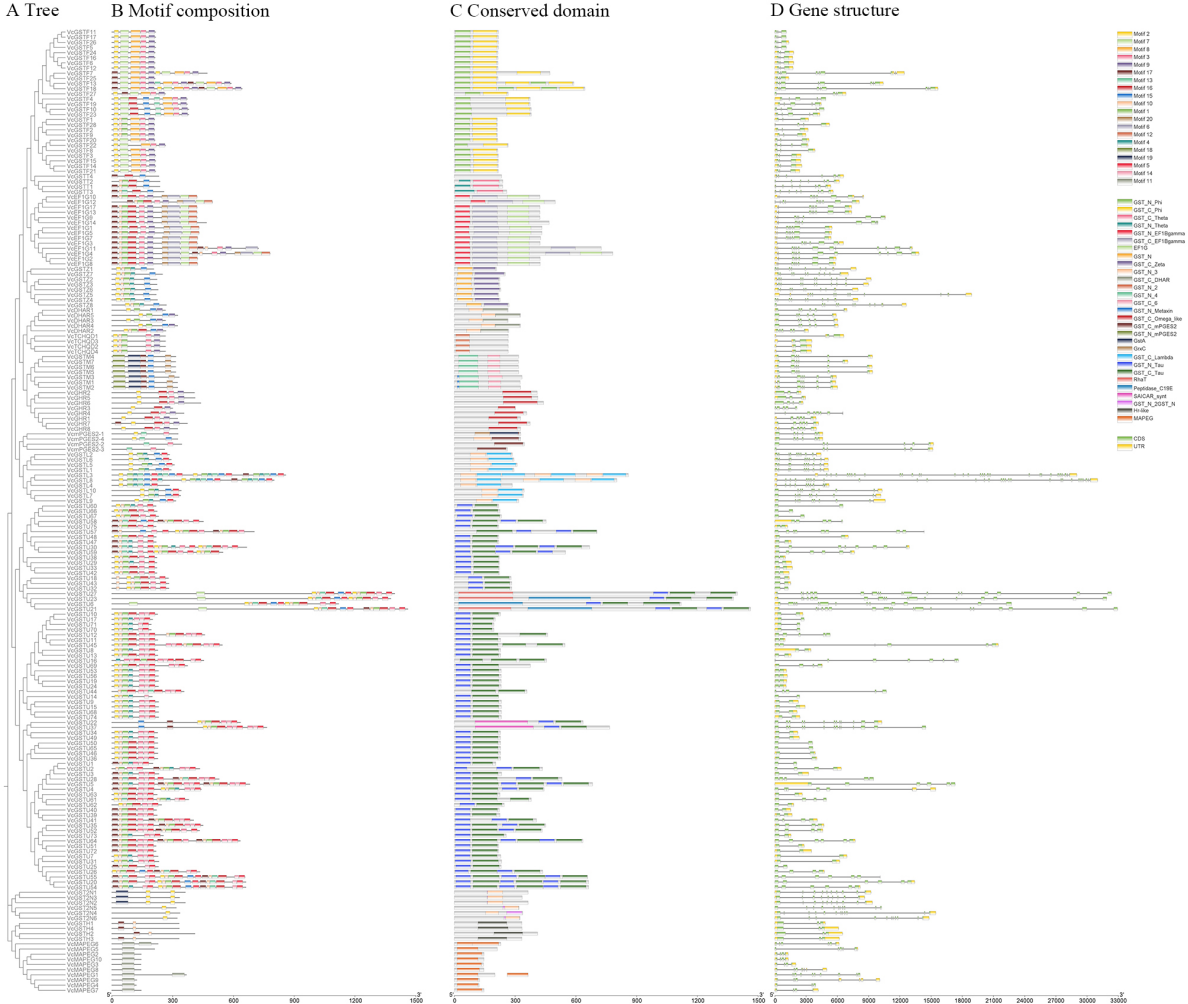
Phylogenetic relationships, conserved motifs, domains, and gene structures of *VcGST* genes. **(A)** Maximum likelihood phylogenetic tree of VcGST proteins. **(B)** Distribution of the twenty conserved motifs in VcGST proteins. **(C)** Conserved domains of VcGST proteins. **(D)** Gene structures of *VcGST* genes, where green boxes represent CDS, yellow boxes represent UTRs, and black lines represent introns.

Analysis of conserved protein structures identified two canonical domains, GST_N and GST_C, in most VcGSTs (**Figure 4**). Notably, some members of the tau and phi subfamilies contained multiple repeats of these domains. In contrast, subfamilies such as GHR, MAPEG, and GST2N lacked one of the two complete domains, possessing only a unique domain structure.

The composition of conserved motifs varied among GST subfamilies. Motifs 1, 2, 4, and 10, corresponding to GST N-terminal domains, were present at least once in each VcGST protein. In contrast, the MAPEG subfamily displayed a distinct motif composition, containing only motif 11. The two largest subfamilies, tau and phi, contained 5–10 conserved motifs, with motif 14 being specific to tau GSTs and motif 8 unique to phi GSTs. Several subfamilies exhibited high internal consistency; for example, all seven metaxin GSTs shared motifs 15, 17, 18, 19, and 20. The conserved motif composition within individual subfamilies, together with clear differences among subfamilies, is consistent with the phylogenetic classification of the VcGST gene family.

The number of exons varied among the different subfamilies. Three main types of exon distribution were identified: (I) The same or similar numbers of exons among genes within the same group; (II) The same number of exons in genes within the same group if they have the complete set of conserved domains, or more exons if they have multiple repeated structural domains; (III) Considerable variation in the number of exons within genes in the same group. The *GSTs* in the DHAR, mPGES2, metaxin, H, and TCHQD classes belonged to type I, with 3-6 exons, respectively. Type III *GSTs* included those in the tau (typically 2 exons, up to 20 exons), phi (3, up to 9), EF1G (8, up to 24), and lambda (10, up to 30) classes. Type III *GSTs* included those in the zeta (9–13 exons), GST2N (9–12), GHR (3–8), MAPEG (3–10), and Teata (6–8) classes. Most *GSTs* in the tau and phi categories had only two or three exons, while those in the zeta category (*VcGSTZ5, VcGSTZ13*) contained the highest number of exons. These findings suggest that the exon-intron structure within the same group is relatively conserved and is closely associated with the evolution of the GST gene family.

### 3.5 Analysis of cis-acting elements in VcGST gene promoter region

**Figure 5:**
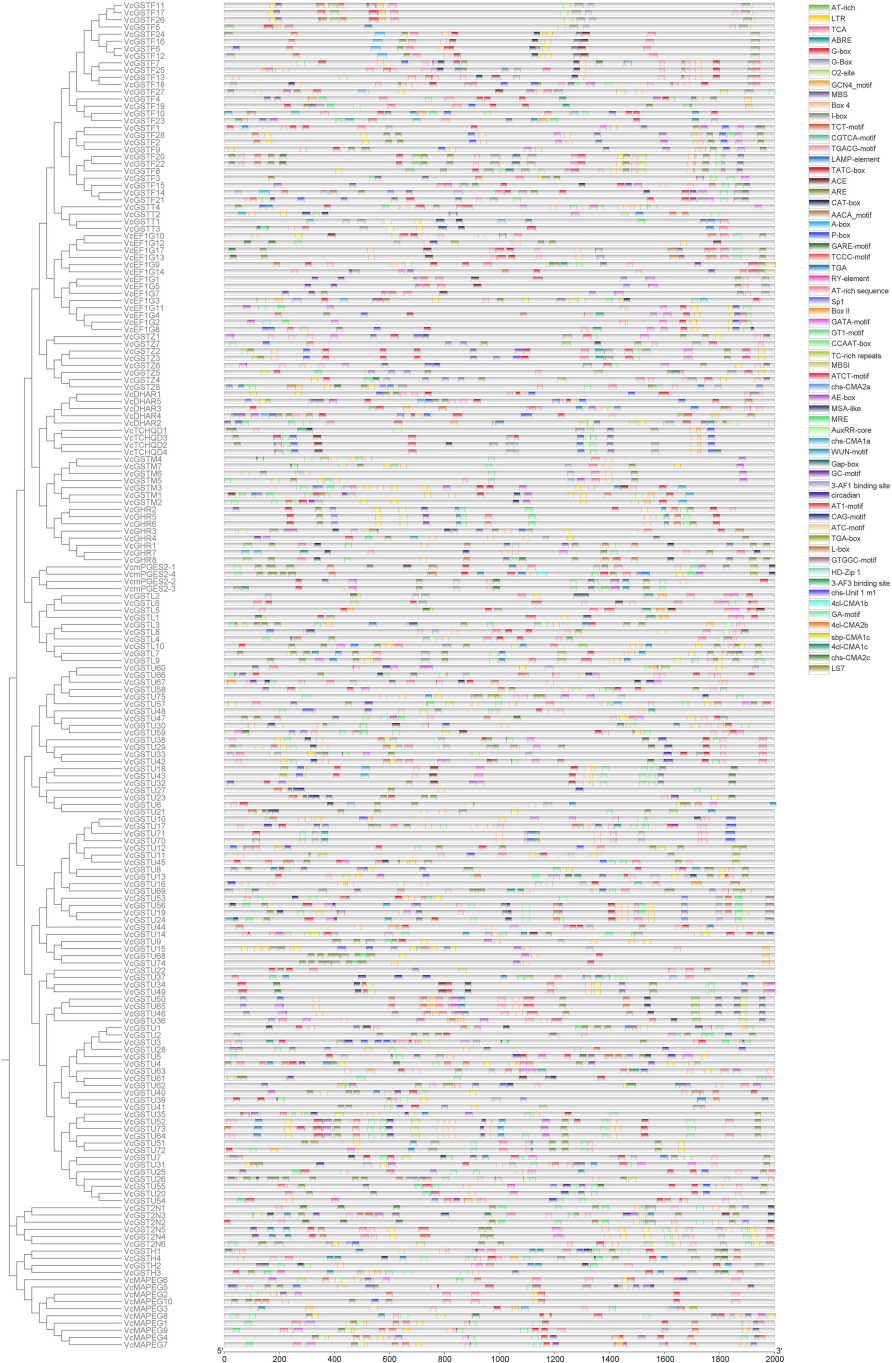
Distribution of predicted cis-acting elements in *VcGST* gene promoters. The predicted cis-elements are represented in boxes of different colors.

The results showed that the cis-acting elements identified in the *VcGST* gene promoter region were classified into four major categories, including light-responsive elements (798), stress-responsive elements (432, oxidative stress, drought, low temperature), hormone-responsive elements (245, methyl jasmonate, abscisic acid, gibberellins, auxin), and elements related to growth and development (157, zein metabolism, meristems, palisade mesophyll cells, endosperm, cell cycle) (**Figure 5**). The stress-responsive elements mainly comprised TC-rich repeats, LTR, ARE, and MBS motifs associated with oxidative stress, drought, and low-temperature responses. These elements were detected in the promoter regions of 146 *VcGST* genes, whereas no such elements were identified in members of the TCHQD subclass. Notably, stress-related cis-elements were more frequently distributed in promoters of *VcGST* genes belonging to the tau and phi classes.In addition, several *VcGST* promoters contained MYB- and MYC-binding sites, which are recognized by MYB and bHLH transcription factors. Among them, MYB-binding motifs previously reported to be associated with anthocyanin biosynthesis were detected in a subset of *VcGST* promoters, implying a potential regulatory association between GSTs and anthocyanin-related processes in blueberry.

Comparative analysis of cis-acting elements in the promoter regions of GST genes from blueberry, Arabidopsis, grape, and apple revealed a largely similar composition of cis-elements among these species (**Supplementary Table 7**). The identified elements were predominantly associated with growth and development, as well as responses to light, stress, and phytohormones. Among them, motifs related to methyl jasmonate (MeJA) responsiveness, abscisic acid responsiveness, and anaerobic induction were the most abundant. These results indicate that GST gene promoters share conserved regulatory features across different plant species, suggesting potential functional conservation of the GST gene family.

### 3.6 Expression profiles of VcGSTs under aluminum stress

**Figure 6:**
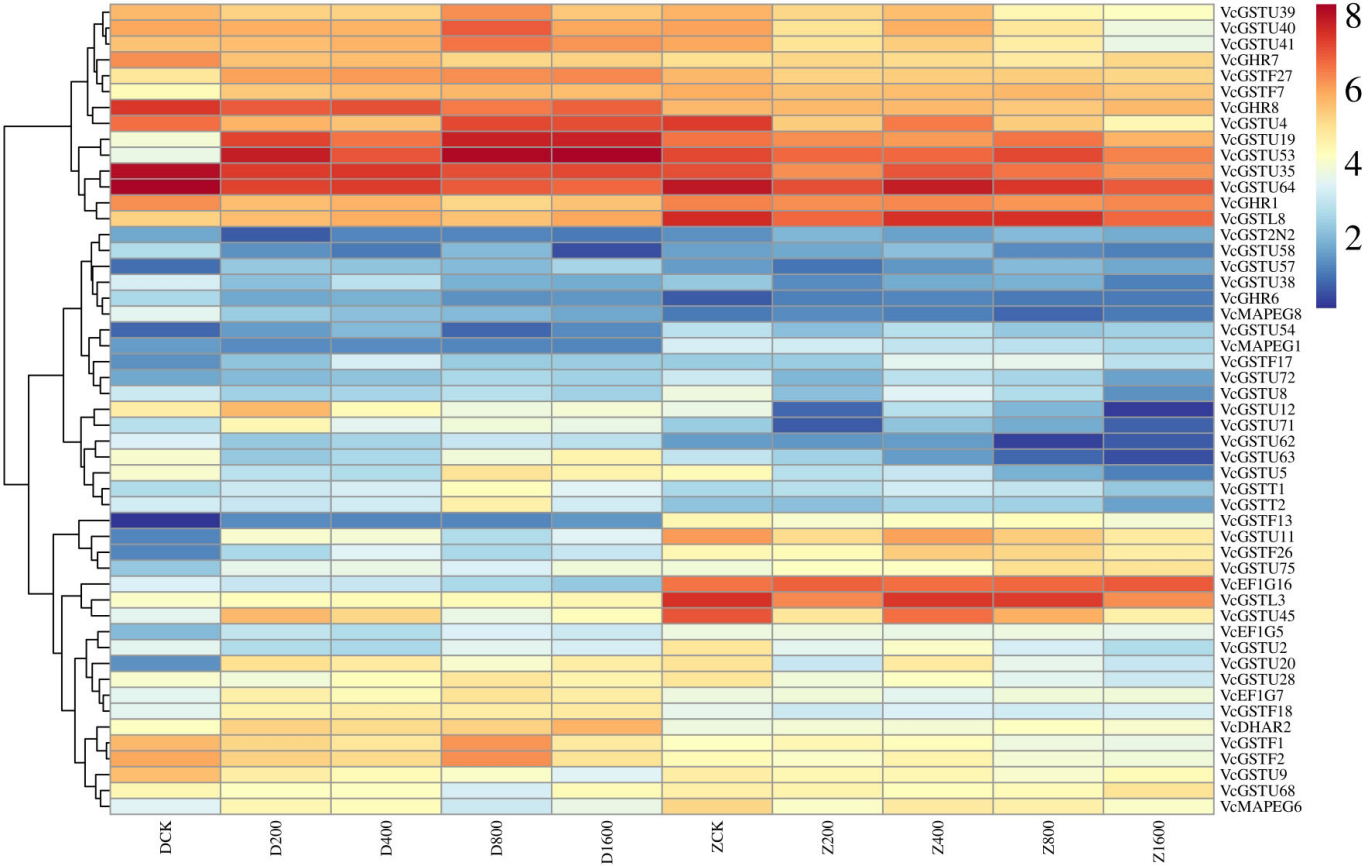
Expression heatmap of VcGST genes in root tissue under aluminum stress.

To identify key VcGST genes responsive to aluminum (Al) stress, transcriptome sequencing was performed on 30 root samples treated with Al. Differential expression analysis based on FPKM values (|log_2_FC| ≥ 1 and FDR < 0.05) revealed 51 differentially expressed VcGST genes (DEGs) in roots under Al stress (**Figure 6**). These DEGs were classified into several GST subfamilies, with the tau (27 genes) and phi (8 genes) classes being the most predominant, followed by GHR (4), EF1G (3), MAPEG (3), lambda (2), theta (2), DHAR (1), and GST2N (1). The significant enrichment of tau and phi class DEGs highlights their potential important roles in mediating Al stress responses in blueberry.

Among all DEGs, *VcGSTU53* (*VaccDscaff35-augustus-gene-220.36*) exhibited the highest transcript abundance and showed a continuous increase in expression under Al stress in the *Vaccinium corymbosum* cultivar ‘Duke’. This gene was annotated as encoding an antioxidant enzyme involved in oxidative stress responses, suggesting a potential function in mitigating Al-induced oxidative damage. Based on its marked upregulation and putative antioxidant function, *VcGSTU53* was selected as the prime candidate for further functional characterization.

The color scale represents log_2_-transformed FPKM values. Rows correspond to genes, and columns correspond to 10 sample group conditions with three biological replicates. Cultivars: D = ‘Duke’; Z = ‘Draper’. Al treatment: 0 (CK), 200, 400, 800, and 1600 μM AlCl_3_·6H_2_O.

### 3.7 Molecular characterization and phylogenetic analysis of VcGSTU53

**Figure 7:**
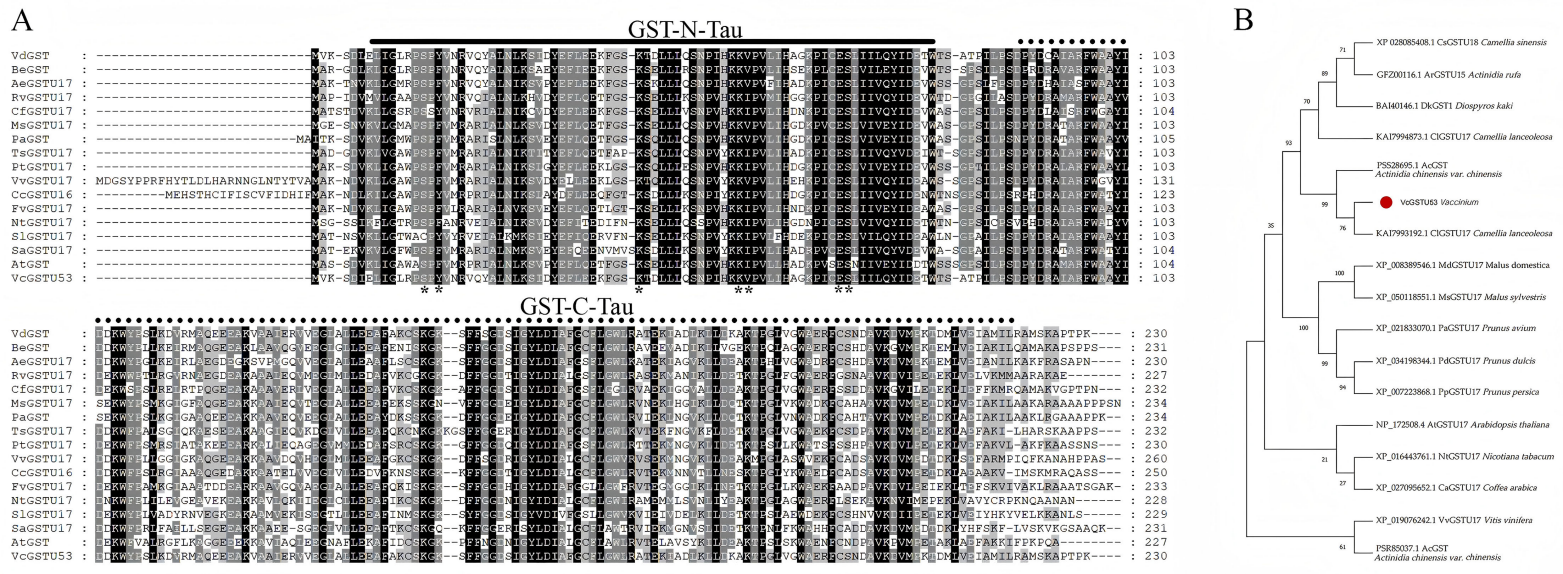
Sequence and phylogenetic analysis of VcGSTU53. **(A)** Multiple sequence alignment of VcGSTU53 with 16 homologous GSTs from other species. **(B)** Phylogenetic tree of 17 GST proteins from different species.

The *VcGSTU53* gene was cloned from aluminum-stressed blueberry roots, with a coding sequence (CDS) of 693 bp, encoding a protein of 230 amino acids (**Figure 7A**). Conserved domain analysis revealed that VcGSTU53 contains the characteristic GST-N-Tau and GST-C-Tau domains, along with seven predicted glutathione (GSH) binding sites. A phylogenetic tree was constructed using VcGSTU53 and its homologs from 16 other plant species (**Figure 7B**). The results showed that VcGSTU53 shares the closest evolutionary relationship with homologs from *Camellia lanceoleosa* (KAI7993192.1, 79.57% identity) and *Actinidia chinensis* var. *chinensis* (PSS28696.1, 76.58% identity). These findings confirm the identity of the cloned gene and provide insight into its evolutionary conservation.

### 3.8 Subcellular localization of VcGSTU53

**Figure 8:**
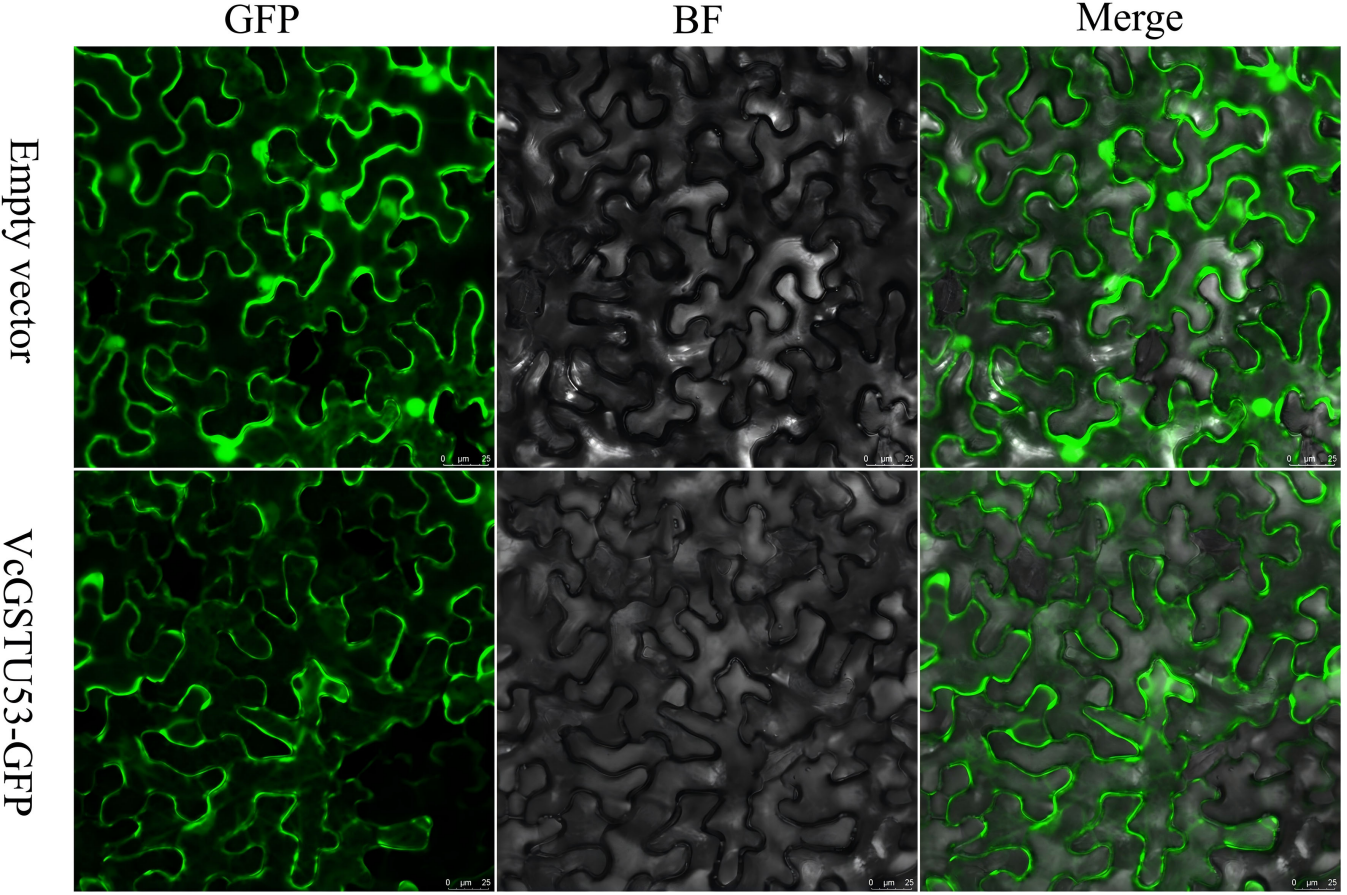
Subcellular localization of VcGSTU53 in the *Nicotiana benthamiana* leaf epidermal cells. Scale bars = 25 μm.

The VcGSTU53 protein was predicted to localize in the cytoplasm by the PROTCOMP online tool. To verify this prediction, a VcGSTU53-GFP fusion protein was transiently expressed in leaves of *Nicotiana benthamiana*. Forty-eight hours after introducing the construct, GFP fluorescent signals were observed under a Leica SP8 laser confocal microscope. As shown in **Figure 8**, the fluorescence signal of the VcGSTU53-GFP recombinant vector was present in the cytoplasm of epidermal cells of *Nicotiana benthamiana*, confirming that VcGSTU53 localizes in the cytoplasm.

### 3.9 Prokaryotic expression and stress resistance of VcGSTU53

#### 3.9.1 Expression, purification and verification of VcGSTU53

The VcGSTU53 gene was optimized for expression in *Escherichia coli* (*E. coli*.) and cloned into the pET-28a(+) vector with a His-tag for protein purification. The recombinant vector was transformed into BL21 (DE3) E. coli and confirmed by PCR. Sequencing confirmed that the inserted gene was VcGSTU53, indicating successful construction of the expression vector.

**Figure 9:**
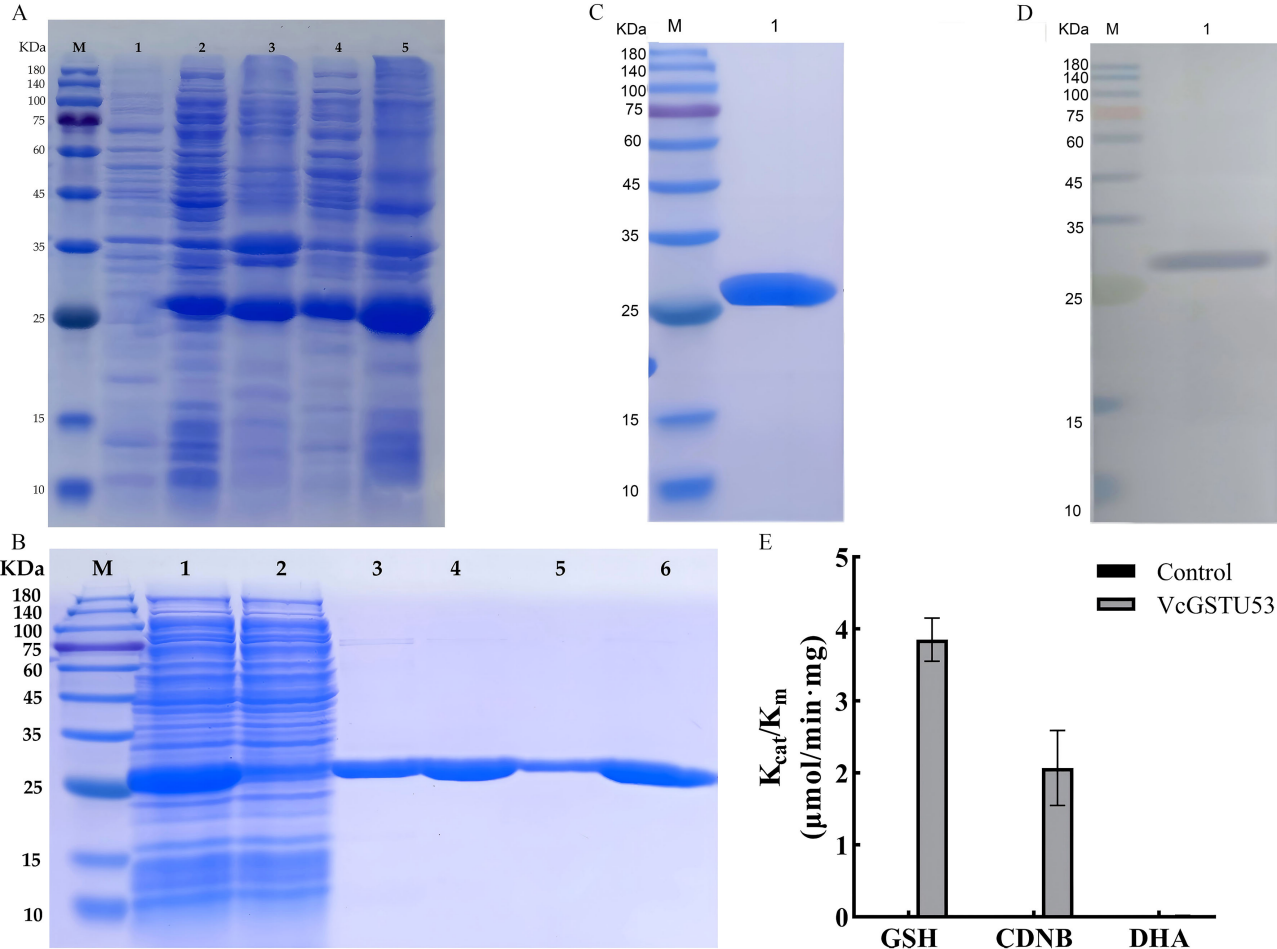
Expression, purification and verification of fusion protein expression. **(A)** SDS-PAGE analysis of fusion protein expression. M: Protein Marker; 1. Total proteins before induction; 2. Supernatant at 20°C; 3. Pellet at 20°C; 4. Supernatant at 37°C; 5. Pellet at 37°C. **(B)** Schematic of purification of fusion protein by nickel agarose affinity chromatography and analysis by SDS-PAGE. M: Protein marker; 1: Loading; 2: Effluent; 3: 20 mM imidazole eluted fraction; 4-5: 50 mM imidazole eluted fraction; 6: 500 mM imidazole eluted fraction. **(C)** SDS-PAGE analysis. **(D)** Immunoblot analysis. **(E)** Determination of VcGSTU53 enzyme activity against three substrates.

Recombinant protein expression was induced under optimal conditions: overnight culture at 20°C, followed by induction with 0.5 mM IPTG and incubation at 37°C for 6 hours. The VcGSTU53×6HIS protein was successfully expressed and purified (**Figures 9A, B**). Elution with 500 mM imidazole yielded the highest concentration of protein (1.8 mg/mL). SDS-PAGE analysis revealed a band corresponding to the expected molecular weight of 27.9 kDa (**Figure 9C**). Immunoblot analysis confirmed the identity of the recombinant protein, detecting a clear band at 27.9 kDa (**Figure 9D**). The purified protein was stored at −80°C for subsequent use.

#### 3.9.2 Determination of VcGSTU53 enzyme activity

The enzyme activity of VcGSTU53 was tested using three substrates: GSH, CDNB, and DHA. As shown in **Figure 9E**, the Kcat/Km values for VcGSTU53 with CDNB, GSH, and DHA were 3.85 ± 0.30, 2.07 ± 0.52, and 0.01 ± 0.01 μmol/min·mg, respectively. These results indicate that VcGSTU53 exhibits GST activity with GSH and CDNB, but not with DHA. The highest reaction rate was observed when GSH was used as the substrate.

#### 3.9.3 Growth curve of VcGSTU53-expressing recombinant bacteria in liquid medium under various stress conditions

**Figure 10:**
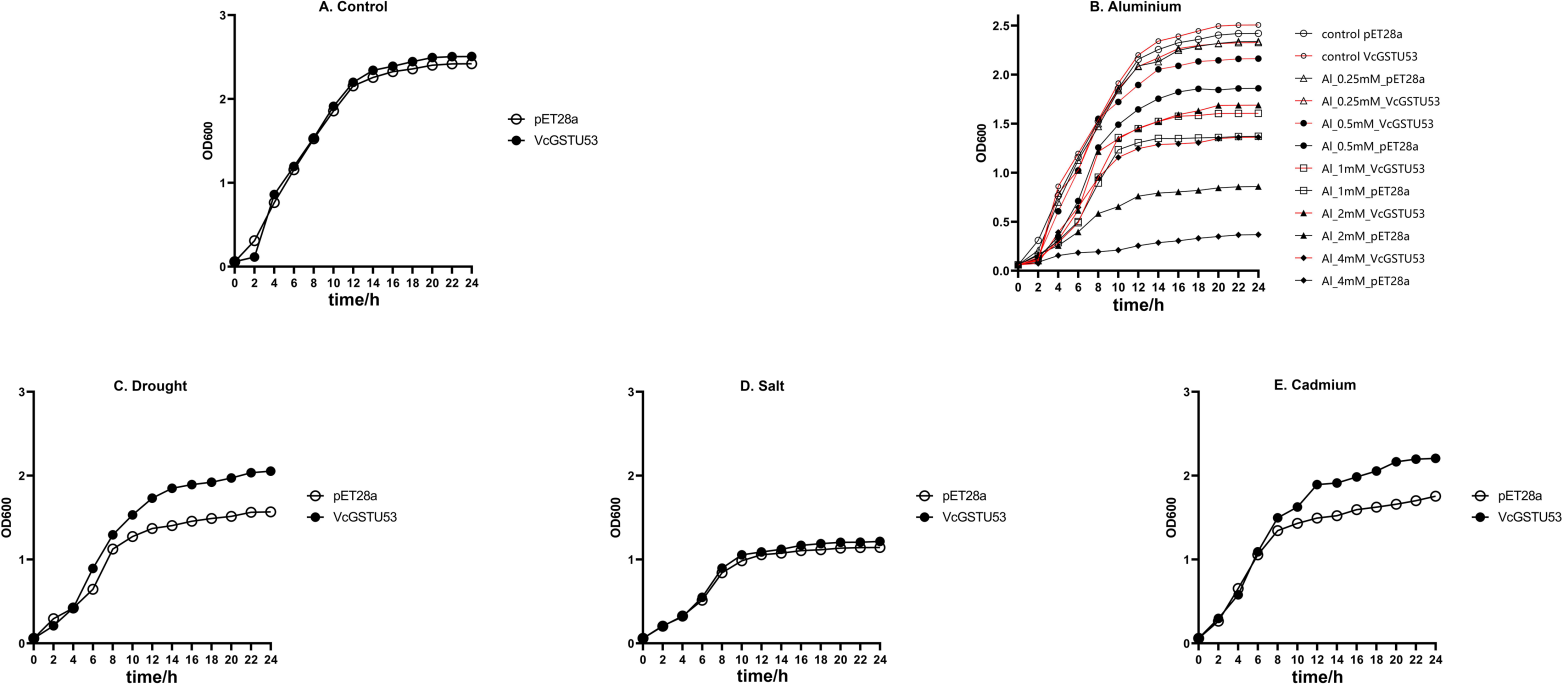
Effect of VcGSTU53 expression on the growth of *E. coli* under various stress conditions. **(A)** Growth curves of the VcGSTU53-expressing strain (VcGSTU53) and the empty-vector control strain (EV) under non-stress conditions. **(B–E)** Comparative growth curves of both strains upon exposure to: **(B)** aluminum (0, 0.25, 0.50, 1, 2, 4 mM AlCl**_3_**·6H**_2_**O), **(C)** drought (1.2 mM PEG8000), **(D)** high salt (500 mM NaCl), and **(E)** cadmium (1 mM CdCl**_2_**).

To evaluate the stress tolerance conferred by VcGSTU53, recombinant *E. coli* expressing VcGSTU53 and the empty-vector control strain were cultured in liquid media containing various stress conditions, and growth was monitored by OD_600_ measurements (**Figure 10**).

Under non-stress conditions, the growth curves of both strains were similar, indicating that expression of VcGSTU53 did not affect normal bacterial growth (**Figure 10A**). In aluminum stress assays, VcGSTU53 conferred significant protection (**Figure 10B**). At the lowest Al^3+^ concentration (0.25 mM), growth was not significantly affected in either strain. However, at concentrations ≥0.5 mM, the VcGSTU53-expressing strain showed significantly better growth than the control. At 4 mM AlCl_3_, only the VcGSTU53-expressing strain survived and grew, while the control strain was completely inhibited.

VcGSTU53 also influenced bacterial tolerance to other stresses differently (**Figures 10C–E**). Under PEG8000-induced osmotic stress, both strains grew similarly for the first 4 hours, after which the VcGSTU53 strain showed a clear growth advantage (**Figure 10C**). In contrast, under high salt stress (500 mM NaCl), the growth of both strains was similar, with OD_600_ of approximately 1.22 before entering the stationary phase, indicating no improvement in salt tolerance due to VcGSTU53 expression (**Figure 10D**). Under cadmium stress (1 mM CdCl_2_), the growth of both strains was similar for the first 6 hours, but the VcGSTU53-expressing strain showed a significantly higher final OD_600_ of 2.21 at 24 hours, compared to 1.76 for the control (**Figure 10E**).

In summary, the *VcGSTU53* gene was identified from transcriptome data associated with aluminum stress and has been shown to enhance aluminum tolerance in *E*. *coli*. Additionally, it has been found that VcGSTU53 responds to other abiotic stresses, including drought and cadmium exposure.

## 4 Discussion

Glutathione S-transferases (GSTs) constitute a multifunctional enzyme superfamily that is widely distributed across animals, plants, and microorganisms, where they play crucial roles in detoxification, redox homeostasis, and stress adaptation (Gullner et al., 2018). Plant GSTs were first identified in maize in 1970, and since then, GST gene families have been systematically characterized in numerous plant species, including Arabidopsis, grape, and apple (Zhao et al., 2021; Jain et al., 2010; Xu et al., 2017; Han et al., 2018; Wang et al., 2018; Wang et al., 2020; Zhuge et al., 2020; Lai et al., 2021). Following the discovery of two novel GST subfamilies, designated hemerythrin (H) and Iota (I), in the non-vascular plant *Physcomitrella patens* (Liu et al., 2013), a total of 14 GST subfamilies have now been recognized in plants (Lallement et al., 2014; Sylvestre-Gonon et al., 2019; Cao et al., 2022). A total of 190 GST genes were identified in the blueberry genome and classified into 14 distinct subfamilies, with the tau (75 members) and phi classes (28 members) being the most abundant. This number substantially exceeds that of typical diploid plants. Notably, no Iota class GST members were identified in blueberry, which is consistent with patterns observed in other higher vascular plants. To date, 13 GST subfamilies have been identified in *Brassica* species among which only the H class is present, while I class GSTs remain absent (Wei et al., 2019). This apparent absence may reflect the loss of I class GST genes during the evolutionary transition from non-vascular to vascular plants. Alternatively, I class GSTs may have undergone extensive sequence divergence, rendering them undetectable using currently available conserved domain models.

The expansion of gene families is generally driven by multiple gene duplication mechanisms, including tandem, segmental, and whole-genome duplications, as well as transpositions. These processes play a central role in plant genome evolution and functional innovation (Flagel and Wendel, 2009). In this study, a total of 190 *VcGST* genes were identified, with WGD and segmental duplication events being the primary drivers of the expansion of the blueberry GST gene family. This result is highly consistent with the allopolyploid genomic background of *Vaccinium corymbosum* (highbush blueberry) and aligns with the common evolutionary pattern observed in polyploid plants, where GST gene families generally expand through large-scale duplications (Jiao et al., 2014; Panchy et al., 2016) In contrast, tandem and proximal duplications contributed less to the overall size of the VcGST family, only playing a role in specific gene clusters.

Most duplicated genes were under purifying selection (Ka/Ks < 1), indicating stable core functions. However, two gene pairs in the Lambda subfamily, VcGSTL7/VcGSTL10 and VcGSTL7/VcGSTL9, showed Ka/Ks values greater than 2, suggesting strong positive selection. By contrast, only one gene pair in the tau subfamily, VcGSTU20/VcGSTU26, had a Ka/Ks value slightly greater than 1 (1.12). These findings strongly suggest that some Lambda subfamily genes have undergone rapid functional divergence after duplication, possibly playing an innovative role in blueberry adaptation to specific environmental stresses. A similar result regarding the lambda subfamily was also reported in *Quercus dentata*, indicating the need for further research into the expansion of this subfamily (Sha et al., 2025).

Promoter cis-element analysis further revealed potential functional diversification of the VcGST family at the transcriptional regulation level. The VcGST promoters contain numerous cis-elements associated with light response, stress response, and hormone signaling. Notably, cis-elements responsive to anaerobic conditions and MeJA were significantly more abundant in blueberry than in Arabidopsis or apple, and were especially enriched in the tau and phi subfamilies. This distribution pattern is consistent with the dominance of tau and phi-class GSTs in family expansion, suggesting their important regulatory role in environmental stress adaptation (Licciardello et al., 2014).

Interestingly, MYB and MYC transcription factor binding sites were detected in the promoters of several VcGST genes. These cis-elements are known to be closely associated with the regulation of anthocyanin biosynthesis and transport (Li et al., 2022). Studies suggest that a conserved “MYB/bHLH-GSTF” regulatory module likely exists in blueberry, where it directly regulates the expression of specific VcGSTF8, thereby mediating the transport of anthocyanins from the cytoplasm to the vacuole and ultimately influencing fruit coloration (Zhang et al., 2024). This mechanism has been validated in Arabidopsis, grape, and other species (Sun et al., 2012; Pérez-Díaz et al., 2016). Thus, the coordinated evolution of gene duplication and regulatory element diversification may have jointly facilitated the functional diversity of the VcGST family in blueberry, contributing to both abiotic stress adaptation and secondary metabolic regulation. Whether the candidate genes are capable of transporting anthocyanins remains to be further investigated.

Highbush blueberry is an acidophilic crop that thrives in soils with a pH range of 4.3–5.5. In Chile, it is typically cultivated in volcanic ash-derived soils with high acidity and aluminum (Al) content (González-Villagra et al., 2021) Despite this adaptation, the molecular mechanisms underlying aluminum tolerance in highbush blueberry remain largely unclear. In this study, VcGSTU53 were identified as a key aluminum-responsive gene. Under normal conditions, VcGSTU53 exhibited low expression in blueberry roots; however, its expression increased more than 30-fold under aluminum stress, surpassing that of other GST family members. Tau-class GSTs are present in all vascular plants and represent the largest GST subfamily. Growing evidence indicates that tau-type GSTs play critical roles in heavy metal stress responses. Transcriptomic and proteomic analyses across multiple plant species, including rice, Arabidopsis, soybean, and citrus, have consistently revealed strong and conserved induction of tau-class GSTs under aluminum stress. In rice, OsGSTU5 mediates arsenic detoxification through glutathione (GSH) conjugation, a process coupled with vacuolar sequestration through the ABC transporter OsABCC1. These findings highlight the conserved function of tau-class GSTs in metal detoxification and intracellular metal compartmentalization.

Consistent with these studies, heterologous expression of VcGSTU53 in *Escherichia coli* (*E. coli*) significantly enhanced aluminum tolerance. This provides functional evidence supporting its role in aluminum resistance. In addition to metal stress, tau-class GSTs are involved in responses to other abiotic stresses. In rice, OsGSTU17 was identified as a positive regulator of drought tolerance, as OsGSTU17 mutants accumulated excessive ROS and exhibited increased sensitivity to water deficit (Li et al., 2023). In soybean and alfalfa, GmGSTU23 and MsGSTU17 enhance salt tolerance by maintaining redox homeostasis and activating antioxidant enzymes (Li et al., 2023; Yu et al., 2025). In *Avena sativa*, several tau-class GSTs are strongly induced by polyethylene glycol (PEG)–mediated drought and cadmium stress. These GSTs are suggested to function through enhanced glutathione metabolism and ROS scavenging (Xu et al., 2024). In this study, overexpression of VcGSTU53 in *E. coli* increased tolerance to cadmium and drought stress, in addition to aluminum stress. This pattern suggests that VcGSTU53 is involved in multiple stress-response pathways. Sequence analysis revealed several predicted GSH-binding sites in VcGSTU53. *In vitro* enzyme assays further confirmed its glutathione transferase activity, using GSH and CDNB as substrates. These results validate its biochemical function as a canonical GST enzyme. Based on these findings, two potential mechanisms may explain the role of VcGSTU53 in aluminum tolerance. First, VcGSTU53 may facilitate metal chelation through GSH conjugation and promote vacuolar sequestration, thereby reducing aluminum toxicity. Second, VcGSTU53 may enhance glutathione-dependent antioxidant capacity and ROS scavenging, contributing to oxidative stress mitigation. Further studies are needed to differentiate between these mechanisms. Taken together, the results position VcGSTU53 as a promising candidate gene for enhancing abiotic stress tolerance in plants. However, its biological function in planta remains to be fully validated, primarily due to technical limitations in achieving stable genetic transformation in blueberry. Future research should prioritize the development of stable transgenic blueberry lines using approaches such as overexpression, CRISPR–Cas or RNA interference. These strategies will facilitate direct functional validation of VcGSTU53 and provide deeper insights into the mechanisms by which tau-class GSTs contribute to abiotic stress adaptation in blueberry.

## 5 Conclusions

A total of 190 *VcGST* genes were identified from the blueberry genome, and were classified into 14 subfamilies: tau (U, 75), phi (F, 28), EF1G (17), lambda (L, 10), MAPEG (10), zeta (8), GHR (8), Metaxin (M, 7), GST2N (6), DHAR (5), hemerythrin (H, 4), mPGES2 (4), TCHQD (4) and theta (T, 4). No iota (I) class *GST* was identified in the blueberry genome. The length of *VcGST* genes ranged from 366 bp to 4386 bp, encoding polypeptides of 121 to 1461 amino acids, with predicted molecular weights between 13.86 KDa and 163.02 KDa, and theoretical isoelectric points ranging from 4.77 to 9.56. Almost half (46.32%) of the VcGST proteins were predicted to localize in the cytoplasm, with the others predicted to localize in the endomembrane system, chloroplasts, nucleus, mitochondria, extracellular space and plasma membrane. (2) The 190 *VcGST* genes were located on all chromosomes except chromosomes 32 and 48 and some scaffolds. Segmental duplications and WGD, followed by tandem duplications, have made the greatest contribution to expansion of the *VcGST* gene family. Analyses of *VcGST* gene promoter regions identified 432 regulatory elements related to stress (e.g., oxidative stress, drought, low temperature), 245 related to hormones (e.g., methyl jasmonate, abscisic acid, gibberellin, auxin) and a large number of binding sites for MYB and BHLH transcription factors. We also identified a binding site for a MYB transcription factor related to anthocyanin synthesis, suggesting that GSTs may be involved in the transport and storage of blueberry anthocyanins. (3) Detailed analysis of *VcGSTU53* revealed that its CDS sequence was 693 bp long, encoding a protein of 230 amino acids. It was differentially expressed in blueberry under aluminum stress, drought stress, and cadmium stress, and among different tissues, and it localized in the cytoplasm. (4) Heterologous expression of *VcGSTU53* in *E. coli* did not affect its normal growth, but increased its tolerance to drought stress, cadmium stress, and especially aluminum stress. *In vitro* enzyme activity experiments confirmed the transferase activity of the VcGSTU53 protein with GSH as the substrate.

## Data Availability

The original contributions presented in the study are included in the article/**Supplementary Material**. Further inquiries can be directed to the corresponding authors.

## Supplementary material

Supplementary Table 1 Identification and classification of GST family of other species.

Supplementary Table 2 The primers used in this study.

Supplementary Table 3 Physicochemical properties and subcellular localization prediction of VcGST.

Supplementary Table 4 Duplication events of *VcGST* genes.

Supplementary Table 5 Collinearity analysis of VcGST family.

Supplementary Table 6 Collinearity analysis of GST gene family among *Vaccinium corymbosum*, *Arabidopsis thaliana* and *Vitis vinifera*.

Supplementary Table 7 Statistical analysis of cis-regulatory elements in the GST promoters of *Vaccinium corymbosum*, *Arabidopsis thaliana*, *Vitis vinifera* and *Malus domestica*.
